# Missing Value Estimation Methods Research for Arrhythmia Classification Using the Modified Kernel Difference-Weighted KNN Algorithms

**DOI:** 10.1155/2020/7141725

**Published:** 2020-06-21

**Authors:** Fei Yang, Jiazhi Du, Jiying Lang, Weigang Lu, Lei Liu, Changlong Jin, Qinma Kang

**Affiliations:** ^1^School of Computer Science and Technology, Shandong University, Qingdao, China; ^2^School of Mechanical, Electrical and Information Engineering, Shandong University, Weihai, China; ^3^School of Computer Science and Technology, Harbin Institute of Technology, Harbin, China; ^4^Department of Educational Technology, Ocean University of China, Qingdao, China; ^5^Department of Computer Science and Technology, Ocean University of China, Qingdao, China; ^6^Beijing Institute of New Technology Applications, Beijing Academy of Science and Technology, Beijing, China

## Abstract

Electrocardiogram (ECG) signal is critical to the classification of cardiac arrhythmia using some machine learning methods. In practice, the ECG datasets are usually with multiple missing values due to faults or distortion. Unfortunately, many established algorithms for classification require a fully complete matrix as input. Thus it is necessary to impute the missing data to increase the effectiveness of classification for datasets with a few missing values. In this paper, we compare the main methods for estimating the missing values in electrocardiogram data, e.g., the “Zero method”, “Mean method”, “PCA-based method”, and “RPCA-based method” and then propose a novel KNN-based classification algorithm, i.e., a modified kernel Difference-Weighted KNN classifier (MKDF-WKNN), which is fit for the classification of imbalance datasets. The experimental results on the UCI database indicate that the “RPCA-based method” can successfully handle missing values in arrhythmia dataset no matter how many values in it are missing and our proposed classification algorithm, MKDF-WKNN, is superior to other state-of-the-art algorithms like KNN, DS-WKNN, DF-WKNN, and KDF-WKNN for uneven datasets which impacts the accuracy of classification.

## 1. **Introduction**

In the present scenario, heart disease is one of the major problems that threaten human health worldwide. Some of them may be indicated by disorders of cardiac rhythm called cardiac arrhythmias. The cardiac arrhythmias can be divided into different types, some kinds of which can cause irreparable long-term damage to the heart, even sudden death [1]. Thus, it is crucial to detect and classify these fatal arrhythmias early, which makes it possible to choose proper antiarrhythmic drugs and give urgent medical treatment.

**Figure 1:**
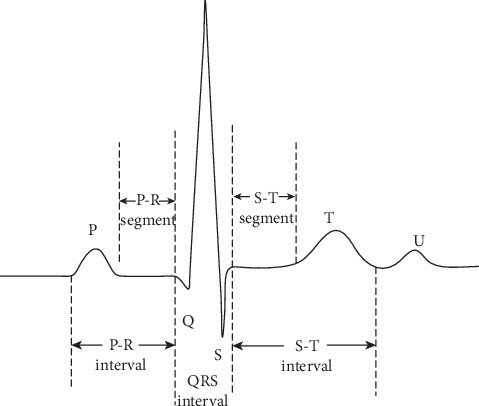
A classic ECG signal sample.

As being noninvasive and easy to record, electrocardiogram (ECG) becomes a preferred diagnostic tool for the detection of arrhythmias and has been broadly used in medical institutes and hospitals. The bioelectrical activity generated by the heart can be recorded and displayed in a graph called the electrocardiograph [2]. One ECG cardiac cycle contains P wave, QRS Complex, and T wave components, which are represented by P, Q, R, S, and T. The main parameters of ECG cardiac cycle for examination of patients with heart disease are amplitude and duration measured according to some characteristics of ECG, i.e., the peak (P, Q, R, S, T, and U), intervals (PR, RR, QRS, ST, and QT), and segments (PR and ST) [3].  Figure 1 shows a classic ECG signal sample that contains the most important features which explore the activity in the heart.

The medical practitioners can interpret the morphology of such an ECG waveform and then detect some irregular changes which are called arrhythmia. However, due to the huge number of patients, the visual checks for arrhythmia are tedious and time-consuming. In addition to it, the conventional manual analysis methods are also usually subjective, which may cause the inaccuracies of the diagnosis results. It is therefore important to apply an automated computing aided approach to detect and classify the arrhythmia more efficiently and precisely. For the last few decades, various techniques have been proposed to assist with the physicians hoping to improve the arrhythmia therapy. In the literature, these processing techniques contain signal processing [4], pattern recognition [5, 6], and machine learning [7–9] methods. This paper is concerned with the imputation methods of the missing values in ECG and the machine learning methods suit for arrhythmia classification.

The remainder of this paper is organized as follows. In Section 2, we briefly discuss the studied problems in the field of arrhythmia classification and review the previous work.  Section 3 describes the arrhythmia dataset, different missing value imputation methods, and our modified algorithm, i.e., MKDF-WKNN.  Section 4 gives the experimental results on the UCI datasets. Finally, the conclusion and future work are drawn in Section 5.

## 2. **Problem Statement**

By far, numerous arrhythmia databases processed by the above techniques have been used as the benchmark by researchers to compare the performance of their research methods with others. Generally, the arrhythmia databases can be classed into two types: signal (e.g., MIT-BIH) and numeric (e.g., UCI). In this paper, we research on the arrhythmia databases that consist of numeric, which have been preprocessed to multidimensional feature vectors by some signal processing and pattern recognition techniques like digital filters and peak analysis [10]. However, some attribute values of the ECG data would inevitably be missing after the preprocessing. Unfortunately, many algorithms for classification like K-Nearest Neighbor (KNN) are not robust to the input matrix with missing values, which may lead to the loss of effectiveness. Therefore, preprocessing before analysis of the data is a crucial task to cope with. For missing values, some studies apply the “case deletion” method directly, i.e., simply removing those instances with missing values and only using the observed instances to establish the classification models, which may lose some information especially for small sample datasets [11]. To tackle these shortcomings, in the past decades, several missing value imputation methods have been proposed in some fields like DNA microarrays [12–16] and traffic data problems [17, 18]. For example, Troyanskaya et al. present a prevalent imputation method based on KNN, i.e., KNN impute for DNA microarrays [13]. Tan et al. propose the PPCA method, a matrix completion method dealing with missing traffic flow problems [18].

The imputation methods have on the whole two divisions which are interpolation based and inductive learning-based methods [18]. The former means to fill the vacancies (missing values) according to the mean or median of the rest values that belong to the same column, or just simply fill them with zeros. Different from the interpolation one, the inductive learning-based methods refer to assign probabilistic values based on the distribution of the known values. For the abovementioned arrhythmia databases that consist of numeric, there is not large literature publishing in missing value imputation. The “case deletion” [19] and “row average” [20] methods are most commonly implemented on the numeric arrhythmia datasets regardless of whether the missing values are significant. These simple approaches are “blind” to the information of the missing values and underestimate the covariance in the data, which may add more uncertainty and lead to bias. In fact, in the previous work, a modified PCA method was proposed to address the missing value problem in the arrhythmia datasets [21]. In this paper, we want to investigate further on a more sophisticated strategy, i.e., Robust Principle Component Analysis (RPCA), an advanced approach. The Matrix Completion (MC) problem can be viewed as a special case of the RPCA problem [22]. The main algorithm of the two ones is the same, and the only difference between the two problems is that the former is to recover the matrix that is corrupted, whereas the latter is to recover the matrix with missing values, so in this paper we can refer to MC method with RPCA method. In MC method, we can solve the optimization problem under some conditions, as in Equation (1), to recover the incomplete matrix [23]:

(1) $$\begin{array}{c} \begin{array}{c} \mathrm{minimize}\mathrm{rank}\left(X\right),\\ \text{subject to}\;X_{ij}=M_{ij},\left(i,j\right)\in \Omega , \end{array} \end{array}$$

where **M** is the observed matrix and the set *Ω* is the indices of **M**. And, this approach is capable to recover matrices of rank about 10 with nearly a billion unknowns from just about 0.4% of their sampled entries [24]. According to this, we also attempt to handle the missing value problem in arrhythmia classification by means of solving this optimization problem and research on the performance of MC for the dataset with different proportions of missing values compared with other state-of-the-art methods for value imputation in arrhythmia classification.

Meanwhile, recent advances in the field of machine learning (ML) for biomedicine and bioinformatics have received considerable attention. These studies are concerned with developing a better and robust automatic algorithm for the classification and identification of the data. In these works, there are some classical machine learning classifiers like Support Vector Machines (SVM), Multilayer Perceptron (MLP), Artificial Neural Network (ANN), K-Nearest Neighbor (KNN), and some variants and combinations of them [25–29], which may make the classification and identification more accurate. Among them, KNN is one of the most commonly used methods for arrhythmia classification for its simplicity and high adaptive behavior [30]. And, some variants of KNN like Distance-Weighted KNN (DS-WKNN) [31] that add weight on distance perform even better than the classical KNN. In our former work, we also propose a kernel Difference-Weighted KNN classifier (KDF-WKNN) [32]. The proposed method improves the accuracy for classification than DS-WKNN.

**Figure 2:**
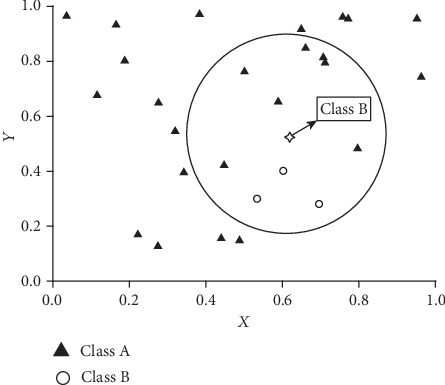
An example of classification in uneven sample.

But for arrhythmia classification or some other multiclass biomedical classifications, they suffer from the unbalanced number of training samples. To illustrate better, we take for example a classification problem in a two-dimensional space to demonstrate the influence on classification results of the unbalanced number of training samples (Figure 2).

As shown in Figure 2, when the classical KNN is applied with the parameter *k* = 10, the sample *x*_*i*_ will be classified into CLASS A because most cases of the 10 nearest neighbors belong to CLASS A. However, if the truth is that the sample *x*_*i*_ belongs to CLASS B, the result of KNN will be a misclassification caused by the unbalance of the number of training sample. And, it is intuitive that the misjudgment may become more serious when the parameter *k* is lager. In order to overcome this problem shown in Figure 2, we take the imbalance of the sample into account and modify our KDF-WKNN and propose a modified kernel Difference-Weighted KNN classifier (MKDF-WKNN). In the method, we first put forward an improved weighting way that imposes a penalty into the weight of the class whose number is large to reduce the impact from the uneven distribution.

As for arrhythmia classification, this paper not only attempt to figure out the missing value problem under different conditions, but consider the uneven sample number of training data in arrhythmia datasets. The main contributions of our work are summarized as follows:

1. For dataset with variant missing values portions (e.g., 10% ~70%), the influence of different missing value imputation methods on the accuracy of selected classifiers is discussed
2. We also modify the previous KDF-WKNN by adding a weight with respect to the sample number, which improves the accuracy for the arrhythmia classification
3. The experimental results on the UCI database indicate that the MC method is fit for the imputation of the missing values in cardiac arrhythmia classification especially when the missing data volume is larger compared with other methods and our proposed MKDF-WKNN algorithm is superior to the previous one for uneven datasets in terms of classification accuracy.

## 3. **Materials and Methods**

### 3.1. Description of Data Set

The standard multivariate ECG dataset taken here is chosen from the Irvine (UCI) cardiac arrhythmias database of the University of California [33].

This database contains 452 instances of samples with 279 attributes, of which the first to 4 attributes refer to the general information of a patient like age and sex, whereas the rest 275 attributes are the numeric features selected from the ECG signal waveform by some signal or pattern process techniques. These 452 participants can be divided into 16 classes according to the ECG data. The first class is “Normal”, and the other 15 classes are “Abnormal”, corresponding to 15 kinds of arrhythmia. A brief description of the 16 classes is given in Table 1. For more details of the data set, please refer to the download website [33].

The UCI cardiac arrhythmia database contains two significant characteristics. First, there are several missing attribute values (about 0.33%). Second, the distribution of class labels is imbalanced. As shown in Table 1, the “Normal” class has 245 instances of samples whereas one of the abnormal classes “Supraventricular Premature Contraction” has only 2 cases. For the first, second, and third-degree atrioventricular block cardiac arrhythmia class, there is even none instance of sample due to the insufficient of the data sample. Note that, certain class with no samples in the training dataset is not in our consideration. We focus on the imbalance of training data rather than solving the missing sample belonging to a certain class.

In this paper, we conduct our research based on these two characteristics and the specific methods will then be introduced in the next section.

### 3.2. PCA Based and RPCA Based Estimation Methods for Missing Values

In this subsection, we give a brief description of the main formulation of the modified PCA and RPCA method for missing value imputation, respectively.

#### 3.2.1. PCA Based Method

In this subsection, we give a brief description of the modified PCA method for missing value imputation. Denote by a training set **X** = {**x**_1_, **x**_2_, ⋯, **x**_*N*_}, where **X**_*j*_ = [**X**_*j*_^(1)^, ⋯,**X**_*j*_^(*d*)^]^*T*^ is a *d*-dimensional vector. The covariance matrix of classical PCA is then defined as:

(2) $$\begin{array}{c} S_{t}=\frac{1}{N}\overset{N}{\underset{j=1}{\sum }}\left(x_{j}-\bar{x}\right){\left(x_{j}-\bar{x}\right)}^{T}=\frac{1}{N}\overset{N}{\underset{j=1}{\sum }}x_{j}{x_{j}}^{T}-{\bar{x}\bar{x}}^{T}, \end{array}$$

where $\bar{x}$ is the mean vector. To mark the missing attribute values in the training set, we use a variable **Z**_*j*_^(*i*)^ to denote whether the value is missing:

(3) $$\begin{array}{c} z_{j}^{\left(i\right)}=\left\{\begin{array}{c} 0,\text{if }x_{j}^{\left(i\right)}\text{ is missing}\\ 1,\text{else} \end{array}\right.. \end{array}$$

Then, the scattering matrix of the training set can be modified by **Z**_*j*_^(*i*)^ as:

(4) $$\begin{array}{c} S_{t}\left(k,l\right)=\frac{1}{N}\overset{N}{\underset{j=1}{\sum }}x_{j}^{\left(k\right)}x_{j}^{\left(l\right)}z_{j}^{\left(k\right)}z_{j}^{\left(l\right)}-{\bar{x}}^{\left(k\right)}{\bar{x}}^{\left(l\right)}, \end{array}$$

where the corresponding mean vector is defined as ${\bar{x}}^{\left(i\right)}=1/N\sum_{j=1}^{N}x_{j}^{\left(i\right)}z_{j}^{\left(i\right)}.$.

Let **W** be the projector obtained by calculating the eigenvector of the above scatter matrix **S**_*t*_, then the reconstructed sample is:

(5) $$\begin{array}{c} X^{\prime }=WY, \end{array}$$

in which **Y** can be obtained by the least square method utilizing **X**, **Z**, and **W**. Please refer to [21] for the details. The reconstructed matrix is the final data with complete values.

#### 3.2.2. RPCA Based Method

In a recent paper [23], Candès and Recht proved that for some positive constant *C*, the matrix of the sample can be completed by solving the optimization problem in Formula (1) if the number of the observed entries, *p*, obeys *p* ≥ *Cn*^6/5^*r*log*n* in which *r* stands for the rank of the matrix and *n* for the maximum of the numbers of rows and columns. Owning to the nonconvexity, Formula (1), an N-P hard problem, is also proved to be approximated by the nuclear norm one as:

(6) $$\begin{array}{c} \begin{array}{c} \mathrm{minimize}\;{\left\|X\right\|}_{*},\\ \text{subject to}\;X_{ij}=M_{ij},\left(i,j\right)\in \Omega . \end{array} \end{array}$$

There are several state-of-the-art algorithms that can approximately solve the MC problem in Formula (6) to recovery a low-rank matrix including the Accelerated Proximal Gradient (APG) approach [34] and the Singular Value Thresholding (SVT) approach [24]. In this paper, the SVT approach, an easy-to-implement and effective algorithm, is applied through which the MC problem can be further modified as:

(7) $$\begin{array}{c} \begin{array}{c} \mathrm{minimize}\;\tau {\left\|X\right\|}_{*}+\frac{1}{2}{\left\|X\right\|}_{F}^{2},\\ \text{subject to}\;\Gamma_{\Omega }\left(X\right)=\Gamma_{\Omega }\left(M\right), \end{array} \end{array}$$

where *τ* is a large positive scalar and the Γ_*Ω*_(**X**) in Formula (7) is the orthogonal projector onto the input matrix outside of *Ω* so that the (*i*, *j*)*th* component of Γ_*Ω*_(**X**) is equal to the constraints in Formula (6), i.e., **X**_*ij*_ if (*i*, *j*) ∈ *Ω* and zero otherwise. And when *τ* is large enough, the solution of the modified objective function converges to that of Formula (6) after the implementation of the shrinkage iteration by the SVT algorithm. Introducing an intermediate **Y**^*k*^ that starts with **Y**^0^ = 0, the mentioned algorithm inductively is defined as:

(8) $$\begin{array}{c} \left\{\begin{array}{c} X^{k}=\Xi_{\tau }\left(Y^{k-1}\right),\\ Y^{k}=Y^{k-1}+\delta_{k}\Gamma_{\Omega }\left(M-X^{k}\right), \end{array}\right. \end{array}$$

until a stopping criterion is reached. In Formula (8), {*δ*_*k*_} refers to a sequence of positive step sizes. Given the Singular Value Decomposition (SVD) of **X** = **UΣV**^∗^, **Σ** = diag({*σ*_*i*_}_1≤*i*≤*r*_), the soft-thresholding operator *Ξ*_*τ*_ is defined as *Ξ*_*τ*_(**X**)≔**U***Ξ*_*τ*_(**Σ**)**V**^∗^, *Ξ*_*τ*_(**Σ**) = diag({(*σ*_*i*_ − *τ*)_+_}), where *t*_+_ is the positive part of *t*. More details for SVT can be found in [24].

### 3.3. Modified KDF-KNN Algorithm

Denote by a training set {(**x**_1_, *y*_1_), ⋯, (**x**_*m*_, *y*_*m*_)}, where **x**_*i*_ ∈ **R**^*d*^ is the *ith* training sample and *y*_*i*_ ∈ {*ω*_1_, ⋯, *ω*_*c*_} is the class label corresponding to **x**_*i*_. Given an unclassified sample **x**, we can find the first *k* nearest neighbors {(**x**_1_^*NN*^, *y*_1_^*NN*^), ⋯, (**x**_*k*_^*NN*^, *y*_*k*_^*NN*^)} by the Euclidean distance metric. In classical KNN, the label of **x** can be assigned by the majority category label of its *k* nearest neighbors.

Because the nearest neighbor close to the unclassified sample should contribute more to classification, a Distance-Weighted KNN (DS-WKNN) is proposed to assign each nearest neighbor a weight *w*_*i*_ according to a function of distance as follows:

(9) $$\begin{array}{c} w_{i}=\frac{d\left(x_{1}^{NN},x_{k}^{NN}\right)-d\left(x^{NN},x_{i}^{NN}\right)}{d\left(x^{NN},x_{k}^{NN}\right)-d\left(x^{NN},x_{1}^{NN}\right)}. \end{array}$$

In our previous work, we take the correlation of different neighbors into account and present a Difference-Weighted KNN, i.e., DF-WKNN and its kernel version KDF-WKNN. In DF-WKNN, the weight assignment can be defined by solving a constrained optimization problem as follows:

(10) $$\begin{array}{c} \begin{array}{c} w=\arg\min\frac{1}{2}{\left\|x-w^{T}X\right\|}^{2},\\ \text{s}.\text{t}.\;\overset{w}{\underset{i}{\sum }}w_{i}=1. \end{array} \end{array}$$

Let *D* = [**x** − **x**_1_^*NN*^, ⋯,**x** − **x**_*k*_^*NN*^]^*T*^. The optimization problem of Formula (10) can be rewritten as

(11) $$\begin{array}{c} \begin{array}{c} w=\arg\min\frac{1}{2}w^{T}{DD}^{T}w,\\ \text{s}.\text{t}.\;\overset{w}{\underset{i}{\sum }}w_{i}=1. \end{array} \end{array}$$

Let **G**^*k*^ = **DD**^*T*^, through a series of mathematical operations, i.e., Lagrange multiplier method and regularization, the problem in Formula (11) can be transformed into the following equation:

(12) $$\begin{array}{c} \left[G^{k}=G^{k}+\eta \text{tr}\left(G^{k}\right)/k\right]w=1_{k}, \end{array}$$

where tr(**G**^*k*^) denotes the trace of the matrix **G**, and *η* = 10^−0^~10^−3^ is the regularization parameter. Finally, the weights **w** of KNNs are determined by solving the linear equation in Formula (12). For the kernel version KDF-WKNN, the method for solving the weights **w** is similar to that of the DF-WKNN described above. Please refer to [31] for the details.

In order to reduce the impact from the imbalance of sample number, we further modified the obtained **w** generated by KDF-WKNN by setting a correction factor *γ* to punish these classes with a large number. Denote by *φ*(*x*) a function to count the number of a certain class, the algorithm of *γ* is inductively defined as:

(13) $$\begin{array}{c} \gamma_{i}=\frac{\log\left(\lambda_{i}+\left(n/\varphi \left(i\right)+\xi \right)\right)}{\log\left(\lambda_{i}+1\right)},i=1,\cdots ,c, \end{array}$$

where *i* refers to the label of certain class belonging to the *c* classes in the training dataset, and *n* stands for the number of training samples. *ζ* is a positive constant parameter which can be justified according to the sample. And *γ*_*i*_ = round(max(*φ*(*i*))/avg(*φ*(*i*))), in which the numerator and the denominator stand for the maximal and average number of all the classes, respectively. Then, the final weight can be written as **w**_*i*_≔**w**_*i*_∗*γ*_*i*_. From Formula (13), it is intuitive that when the number of one class *i* is large, the correction factor *γ*_*i*_ becomes smaller, so that a lower weight will be assigned to the corresponding sample.

## 4. Experimental Results

In this section, we perform comparison experiments with respect to missing value imputation methods and classification methods for arrhythmia datasets with different proportions of missing values (e.g., 10%~70%). All the experiments are carried out with Intel(R) Core(TM) i5-4590 CPU (3.30 GHz) and 32GB RAM under the Matlab2012a programming environment. The dataset comes from the UCI machine learning repository described in Section 3.1, and the different proportions of missing values are generated by computer at random based on the original UCI arrhythmia dataset.

### 4.1. Experimental Procedure

**Figure 3:**
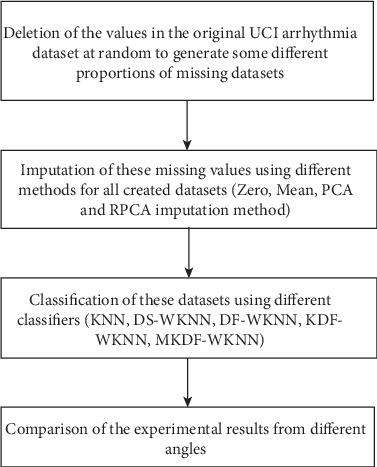
The block diagram of the experimental procedure.

The experimental flow on the UCI arrhythmia dataset is delineated in the flow block diagram (Figure 3), which is comprised of four steps:

1. The first step is to delete different proportion values on the original UCI arrhythmia dataset at random to generate some different proportions of missing value datasets.
2. The second step involves imputation of these missing values using different methods for all created datasets. In our experiment, we apply four methods, i.e., Zero, Mean, PCA, and RPCA imputation methods. “Zero method” means to input the missing values with zero which is used for comparison; “Mean method” refers to replace each missing value with the average value of the corresponding attribute which is commonly applied in arrhythmia classification; The PCA and RPCA method are the two inductive learning-based methods we introduced in Section 3.2.
3. Then, we classify these datasets using different classifiers (KNN, DS-WKNN, DF-WKNN, KDF-WKNN, MKDF-WKNN), and the VALUE of *k* is unified into 151. In our experiment, the value of the parameter in MKDF-WKNN, *ζ*, is 10^−4^. To reduce bias, the performance of these classifiers is evaluated by running 10-fold cross-validation with 9-fold for training and 1-fold for testing. We split the arrhythmia database into 10 folds and the mean classification accuracy is adopted by the average of 10 splits.
4. At last, we compare the performance of these methods for arrhythmia classification and visualize the experiment result.

### 4.2. Classification Performance

In this section, we show the experiment results that were implemented using an accuracy indicator to examine the performance of missing value imputation methods and five classifiers for classifying cardiac arrhythmia. The experiments compare the performance on different proportions of missing value datasets generated from the UCI arrhythmia database. And the whole result is shown in Table 2.

**Figure 4:**
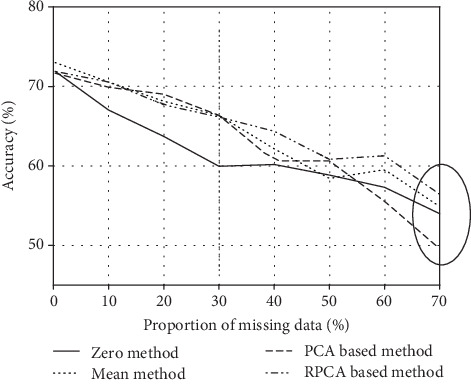
Comparison of the classification accuracy (%) of MKDF-WKNN using four missing value imputation methods for different proportions of missing data.

From Table 2, we can infer that out of five different classifiers the MKDF-WKNN model gives very attractive classification results in terms of classification accuracy through the four missing value imputation methods of 71.90%, 73.01%, 71.68%, and 71.90%, respectively. To further research on the influence of different missing value imputation methods on the accuracy of certain classifiers and observe the trends obviously, we take the MKDF-WKNN classifier as an example and visualize the last four rows of Table 2 in form of line chart (Figure 4).

We empirically tested 7 simulations based on the dataset with the percentage of missing values range from 10% to 70% (the proportion of missing data in the original UCI dataset is 0.33%) using four methods to estimate the missing values, and the accuracy is obtained by the classification on the data after imputation.

Form Figure 4, as we imagine, the accuracy becomes lower and lower with the increase of the percentage of missing values for all the four broken lines. And, when the proportion of missing data ranges from 0% to 30%, the last three imputation methods are similar and perform better than the “Zero method”. For the proportion of missing data ranging from 30% to 70%, the “RPCA-based method” outperforms the other three; however, the “PCA-based method” sharply gets worse, which indicates the lack of stability of the “PCA-based method”. Throughout the whole picture, the “RPCA-based method” is more stable and accurate particularly for the data including a larger number of missing values, which indicates that the “RPCA-based method” can successfully handle missing values in arrhythmia dataset no matter how many values in it are missing. And the “Mean method”, a most commonly used imputation method in arrhythmia classification, is second only to it.

Based on the experimental results, we conclude that for arrhythmia classification, when the small part (0% to 30%) of data is missing, we can apply any of the other three imputation methods except the “Zero method”. And when the missing values are large (30% to 70%), we can use the “RPCA-based method” to replace the classical method, i.e., the “Mean method”.

**Figure 5:**
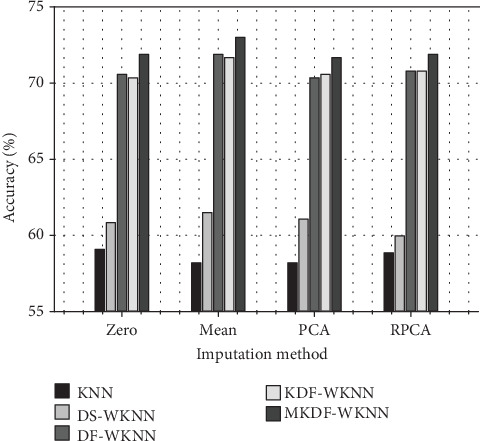
Comparison of the average classification accuracy (%) of KNN, DS-WKNN, DF-WKNN, KDF-WKNN, and MKDF-WKNN.

What is more, we also make a histogram of one column in Table 2 when 0.33% values of the data are missing (the original UCI arrhythmia dataset) in Figure 5.

Figure 5 illustrates the mean classification accuracy of five classifiers, i.e., KNN, DS-WKNN, DF-WKNN, KDF-WKNN, and MKDF-WKNN with four missing value estimation methods. Obviously, we can see that whatever method is chosen to fill the missing data, MKDF-WKNN outperforms the others in terms of classification accuracy, which indicates that the modification is effective for the uneven dataset like the UCI arrhythmia dataset in which the major class labels are “Normal”. In addition, we can infer that the mean accuracy is more than 70% when using our MKDF-WKNN classifier, whereas the mean accuracy of the traditional KNN is lower than 60%, which implies that our proposed algorithm is reliable for the classification of different arrhythmia types so that the problem that stated in Section 2 can be solved.

## 5. Conclusion

Missing value is a crucial problem, which could compromise the quality of data, so missing value estimation is a significant preprocessing step for further experiments. In this paper, we compare the main methods for estimating the missing values in electrocardiogram data like the “Zero method”, “Mean method”, “PCA-based method”, and “RPCA-based method”. In our comparative study, the “RPCA-based method” can successfully handle missing values in the arrhythmia dataset no matter how many values in it are missing, which indicates that the higher classification accuracy can be expected in the practical application when a large number of values in the dataset are missing. As for the imbalance data classification problem, we also propose a modified KNN-based classification algorithm, i.e., MKDF-KNN, which is modified by a correction factor to handle the imbalance datasets problem to get better performance. In the future, we will further study the modified factor for the weight of the weighted-KNN and improve the robustness of the method of selecting better parameters of our MKDF-WKNN.

## Figures and Tables

**Table 1:** Class description of the UCI cardiac arrhythmia database.

Class	Class name	Number of instances
1	Normal	245
2	Ischemic changes (coronary artery disease)	44
3	Old anterior myocardial infarction	15
4	Old inferior myocardial infarction	15
5	Sinus tachycardia	13
6	Sinus bradycardia	25
7	Ventricular premature contraction (PVC)	3
8	Supraventricular premature contraction	2
9	Left bundle branch block	9
10	Right bundle branch block	50
11	1. degree atrioventricular block	0
12	2. degree AV block	0
13	3. degree AV block	0
14	Left ventricular hypertrophy	4
15	Atrial fibrillation or flutter	5
16	Others	22

**Table 2:** Comparison of the classification accuracy (%) achieved w.r.t. the imputation methods and classifiers.

Proportion		0.3%	10%	20%	30%	40%	50%	60%	70%
KNN	X0	59.07	55.75	54.65	54.87	53.98	54.20	53.98	54.42
	MEAN	58.19	57.08	55.53	55.75	54.42	53.98	53.76	54.65
	PCA	58.19	57.96	55.53	56.19	54.65	54.65	53.76	53.76
	RPCA	58.85	57.52	56.86	57.30	55.09	54.42	54.42	55.09

DS-WKNN	X0	60.84	59.96	55.75	54.42	52.88	53.76	52.43	51.99
	MEAN	61.50	59.29	58.19	56.42	55.31	54.20	53.54	53.32
	PCA	61.06	60.18	58.41	58.63	55.31	55.31	53.32	50.88
	RPCA	59.96	58.63	58.63	59.29	54.20	55.53	57.08	54.42

DF-WKNN	X0	70.58	66.15	63.27	59.73	58.85	56.86	56.19	54.20
	MEAN	71.90	69.03	68.14	65.49	61.73	59.29	59.29	56.19
	PCA	70.35	69.03	66.59	65.49	61.28	61.28	55.53	50.44
	RPCA	70.80	67.92	65.93	65.49	62.17	61.06	60.40	57.08

KDF-WKNN	X0	70.35	66.15	63.27	60.18	58.19	56.86	56.19	54.65
	MEAN	71.68	69.03	67.92	65.71	61.73	59.29	59.29	56.19
	PCA	70.58	68.81	67.04	65.93	61.50	61.50	55.09	51.77
	RPCA	70.80	67.70	65.93	64.60	61.95	61.73	60.18	56.86

MKDF-WKNN	X0	71.90	67.04	63.72	59.96	60.18	58.85	57.30	53.98
	MEAN	73.01	70.58	68.14	66.37	62.17	58.41	59.51	54.87
	PCA	71.68	69.91	69.03	66.37	60.62	60.62	55.53	49.56
	RPCA	71.90	70.58	67.70	66.15	64.38	60.84	61.28	56.42

## Data Availability

The processed data are available from the corresponding author request.
